# Transcriptomic Analysis of Dysregulated Genes of the nDNA-mtDNA Axis in a Mouse Model of Dilated Cardiomyopathy

**DOI:** 10.3389/fgene.2022.921610

**Published:** 2022-06-08

**Authors:** Mark Ziemann, Wei Wu, Xiu-Ling Deng, Xiao-Jun Du

**Affiliations:** ^1^ School of Life and Environmental Sciences, Deakin University, Geelong, VIC, Australia; ^2^ Key Laboratory of Environment and Genes Related to Diseases, Department of Physiology and Pathophysiology, School of Basic Medical Sciences, Ministry of Education, Xi’an Jiaotong University Health Science Center, Xi’an, China; ^3^ Baker Heart and Diabetes Institute, Melbourne, VIC, Australia

**Keywords:** transcriptome analysis, nuclear DNA, mitochondrial DNA, mitochondrial RNA, mitoribosome, dilated cardiomyopathy

## Abstract

**Background:** Mitochondrial dysfunction is implicated in the development of cardiomyopathy and heart failure. Transcription of mitochondrial DNA (mtDNA) encoded genes and subsequent protein synthesis are tightly regulated by nuclear DNA (nDNA) encoded proteins forming the nDNA-mtDNA axis. The scale of abnormalities in this axis in dilated cardiomyopathy (DCM) is unclear. We previously demonstrated, in a mouse DCM model with cardiac Mst1 overexpression, extensive downregulation of mitochondrial genes and mitochondrial dysfunction. Using the pre-acquired transcriptome sequencing database, we studied expression of gene sets of the nDNA-mtDNA axis.

**Methods:** Using RNA-sequencing data from DCM hearts of mice at early and severe disease stages, transcriptome was performed for dysregulated nDNA-encoded gene sets that govern mtDNA transcription and *in situ* protein synthesis. To validate gene data, expression of a panel of proteins was determined by immunoblotting.

**Results:** Relative to littermate controls, DCM hearts showed significant downregulation of all mtDNA encoded mRNAs, as well as mtDNA transcriptional activators. Downregulation was also evident for gene sets of mt-rRNA processing, aminoacyl-tRNA synthases, and mitoribosome subunits for *in situ* protein synthesis. Multiple downregulated genes belong to mitochondrial protein-importing machinery indicating compromised importing of proteins for mtDNA transcription and translation. Diverse changes were genes of mtRNA-binding proteins that govern maturation and stability of mtDNA-derived RNAs. Expression of mtDNA replicome genes was largely unchanged. These changes were similarly observed in mouse hearts at early and severe stages of DCM.

**Conclusion:** Transcriptome revealed in our DCM model dysregulation of multiple gene sets of the nDNA-mtDNA axis, that is, expected to interfere with mtDNA transcription and *in situ* protein synthesis. Dysfunction of the nDNA-mtDNA axis might contribute to mitochondrial dysfunction and ultimately development of DCM.

## Introduction

Dilated cardiomyopathy (DCM) and heart failure (HF) constitute the major challenge to modern cardiology. The mortality is high after confirmation of the diagnosis of DCM or HF despite routine medications, reflecting our limited understanding on the fundamental mechanisms underlying the initiation and progression of disease (Murphy et al., 2016; Brown et al., 2017). Mitochondrial dysfunction has been implicated as a common and fundamental abnormality of DCM and HF facilitating the progress of disease (Murphy et al., 2016; Brown et al., 2017). Numerous studies have revealed mitochondrial dysfunction in the failing heart manifested as lower energy generation mismatching the demand, increased generation of reactive oxygen species, and cell death mediated by mitochondrial death signaling (Murphy et al., 2016; Brown et al., 2017). Whereas the molecular mechanism responsible for mitochondrial dysfunction remains unclear, recent studies have demonstrated that activation of the Hippo signaling pathway is potent in mediating extensive suppression of mitochondrial genes together with significant mitochondrial dysfunction leading to DCM (Liu et al., 2021; Wu et al., 2021).

In mammalian cells, mitochondrial DNA (mtDNA) is a small (approximately 16,000 bp), circular, double-stranded molecule residing in mitochondrial matrix (Murphy et al., 2016). mtDNA encodes 13 mRNA for polypeptides as components of oxidative phosphorylation (OXPHOS) complexes, in addition to tRNAs and rRNAs (Kummer and Ban, 2021). RNAs derived from mtDNA are subsequently undergone modifications, maturation or degradation by proteins that bind to specific motifs (Barchiesi and Vascotto, 2019; Ravanidis and Doxakis, 2020). Normal function of transcription/translation of mtDNA-encoded mRNAs is dependent on multiple interrelated machineries forming the nuclear DNA (nDNA)-mtDNA axis, that govern transcriptional regulation of mtDNA genes (Kummer and Ban, 2021), protein synthesis *via* mitochondrial ribosome (mitoribosome) (Kummer and Ban, 2021), and mitochondrial protein importing system that transports, modifies, localizes and assembles 99% of mitochondrial proteins (Kummer and Ban, 2021). Accordingly, faulty in mtDNA transcription and translation would be expected to contribute to the development of cardiomyopathy and HF. Research has also documented abnormalities in transcription of mtDNA-encoded mRNAs as well as tRNA and rRNA in the disease heart (Barchiesi and Vascotto, 2019). Such abnormalities are considered to influence assembly of complexes localized in the inner mitochondrial membrane (IMM) (Murphy et al., 2016; Brown et al., 2017; Pfanner et al., 2019). However, it remains poorly understood on the transcriptional dysregulation of the genes of the nDMA-mtDNA axis in the setting of cardiomyopathy.

Our recent study showed that cardiomyocyte-restricted overexpression of Mst1 (mammalian sterile 20-like kinase) leads to activation of the Hippo signaling pathway with extensive downregulation of multiple gene sets related to mitochondrial turnover and metabolism (Wu et al., 2021). By employing transcriptome sequencing from Mst1-TG model, we conducted a detailed transcriptome analysis on changes not only in mtDNA-encoded genes, but also in several gene sets for proteins that form the nDNA-mtDNA axis participating in mtDNA transcription and translation. We observed dysregulation in multiple gene sets that are expected to influence the operation of nDNA-mtDNA axis in this DCM model.

## Methods

### Animals and RNA Sequencing

We previously performed RNA-sequencing of left ventricular tissues from transgenic strain of mice with cardiac overexpression of Mst1, an upper-stream kinase of Hippo pathway (Yamamoto et al., 2003). The use of the α-myosin heavy chain promoter (α-MHC) allows for cardiomyocyte-restricted overexpression of Mst1 that drives the onset of DCM as thoroughly documented by our previous studies (Nguyen et al., 2019; Wu et al., 2021). Male Mst1-TG (DCM) and non-TG littermate (nTG) mice were used.

Heart tissues from nTG and Mst1-TG mice were used for RNA-sequencing and immunoblotting. Animals were studied at a young (3 weeks) and adult (15 weeks) ages, representing early or severe stage of DCM, as being demonstrated previously (Nguyen et al., 2019; Wu et al., 2021). Left ventricular tissues were homogenized in TRIzol and RNA isolation was done using RNA Miniprep Kit (Zymo Research), and transcriptome sequencing of extracted RNA was performed. Details of transcriptome sequencing were provided in our previously published studies (Nguyen et al., 2019; Wu et al., 2021).

### Transcriptome Analysis

Fastq files underwent quality trimming of low quality bases from the 5′ end of reads using a minimum phred threshold of 10, followed by mapping to the mouse Gencode v24 transcriptome (Frankish et al., 2019) with Kallisto (Bray et al., 2016). Transcript counts were read into R v4.1.3 and these were aggregated to gene-level counts. Genes with fewer than 10 reads per sample on average were discarded. DESeq2 v1.32.0 was used for differential expression analysis (Love et al., 2014). Pathway analysis was conducted using mitch v1.4.1 (Kaspi and Ziemann, 2020) using the default settings and gene sets from Reactome obtained on the 16th February 2022 (Gillespie et al., 2022). By default, mitch does not apply a fold-change threshold. As a functional class scoring method, it requires input data for all detected genes. By default, mitch ranks each gene based on the Wald test statistic provided by DESeq2 which is a type of directional significance value. Genes and gene sets with a false discovery rate (FDR) adjusted *p*-value < 0.05 were considered statistically significant. Heatmaps were generated with the heatmap.2 function.

### Immunoblotting

Protein immunoblotting was performed as we previously described (Wu et al., 2021). Using LV tissues from mice aged at 3 and 15 weeks, protein was extracted by homogenization of tissues with lysis buffer containing proteinase inhibitor cocktail (Sigma), and phosphatase inhibitor cocktail (Sigma). Protein (20–30 μg) together with molecular markers (Thermo) was separated on 4%–15% MINI-protean tgc-Stain free gels (BioRad), and then transferred onto a polyvinylidene membrane (Millipore). After blotting with 5% skim milk in Tris-buffered saline-Tween 20, the membrane was incubated overnight (4°C) with primary antibodies against selected proteins according to the manufacturers’ instructions, followed by incubation with a secondary antibody. The membrane was blocked and protein bands were visualized by enhanced chemiluminescence using Clarity™ Western ECL Substrate (Bio-Rad). Normalization of target proteins was made with GAPDH or β-actin as a housekeeping proteins detected using the same membrane. Commercial source and concentration of antibodies against selected proteins were as follows: mtCO2 (Abcam, 1:1000), mtND1 (Abcam, 1:5000), Tfam (Abcam, 1:2000), Lrpprc (Abcam, 1:1000), Rnase4 (Abcam, 1:500), Mfn1 (Abcam, 1:1000), Twnk (Abcam, 1:1000), Polrmt (Invitrogen, 1:1000), Mrpl45 (Thermo, 1:1000), PGC-1α (Thermo, 1:1000), VDAC1 (Thermo, 1:4000), GAPDH (Proteintech, 1:4000), and β-actin (Proteintech, 1:4000).

## Results

### Overall RNA-Sequencing Findings

**FIGURE 1:**
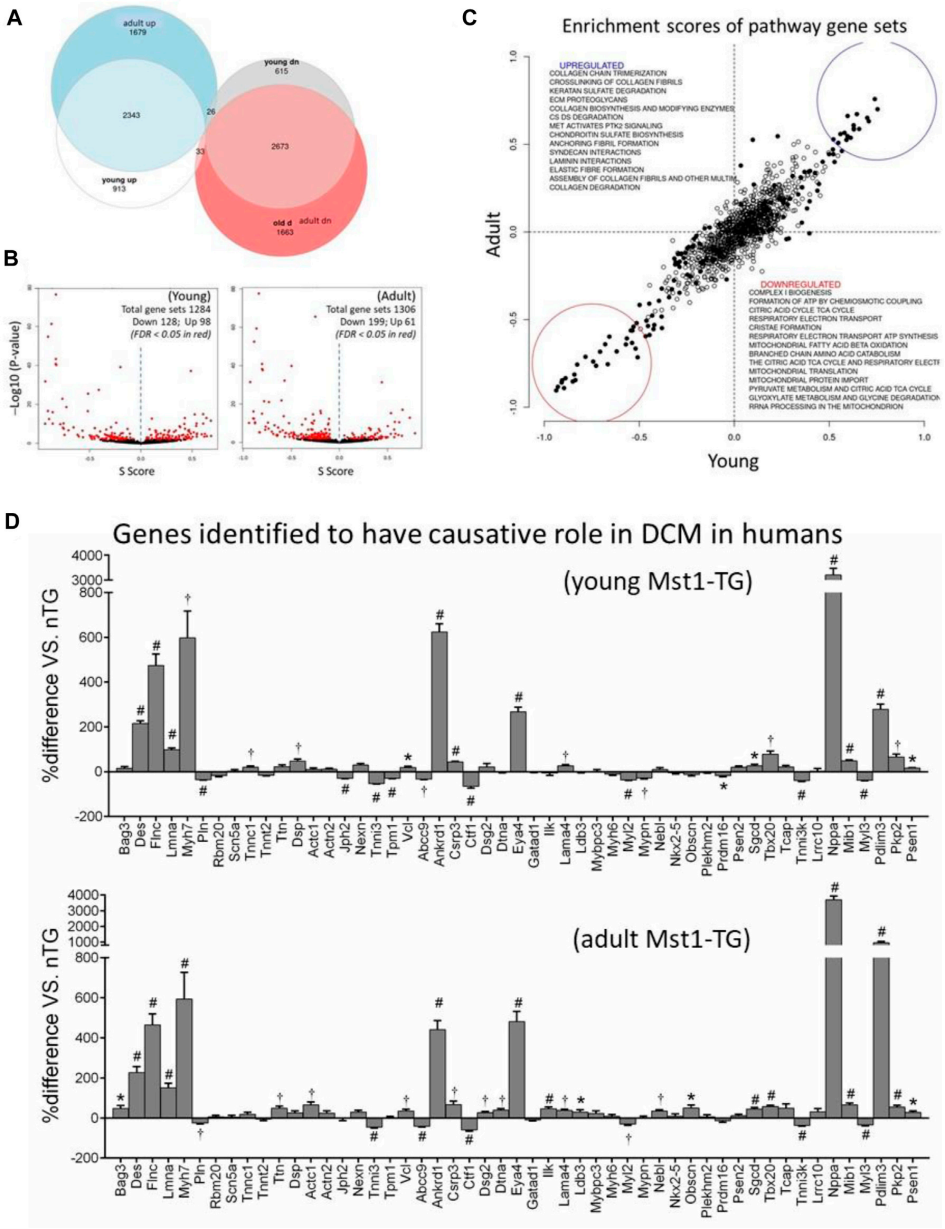
Differentially expressed genes and gene sets by transcriptome profiling in DCM hearts. Cardiotranscriptome profiling of left ventricular tissues from young (3 weeks, *n* = 4 per genotype) and adult (15 weeks, *n* = 6 for nTG control and *n* = 7 for DCM) mice. **(A)** Euler diagram for the differentially expressed genes in young and adult DCM hearts. **(B)** Volcano plots of differentially expressed gene sets. Each dot represents one gene set with set size ranging from 10 to 1697; **(C)** Scatterplot of Enrichment scores of pathway gene sets showing consistency in up or downregulated gene sets between young and adult DCM hearts relative to respective control. Pearson R = 0.91 and Spearman R = 0.85. **(D)** Expression of 51 genes implicated as causal genes for human DCM. *p < 0.05, ^†^ *p* < 0.01, #*p* < 0.001 vs. nTG.

There were 4 samples for control and DCM groups for young age, and 6 samples for control and 7 samples for DCM groups of the adult age. We generated 25 M (young) or 38.6 M (adult) reads per sample, of which 83.3% of reads were uniquely mapped (SD 2.1%). An average of 24.0 M reads was assigned to genes (SD 3.2 M) yielding about 50,000 genes present in the annotation set. Approximately 15,000 genes were expressed above the detection threshold of 10 reads per sample. A total of 14,888 genes were similarly identified in both age groups. As we previously described (Nguyen et al., 2019; Wu et al., 2021), relative to control hearts, differentially expressed genes (DEGs; false discovery rate <0.05) accounted for 43.2% (*n* = 6,603) and 41.7% (*n* = 8,417), respectively, of total genes in young and adult DCM hearts. There were 2,343 DEGs and 2,673 DEGs that were similarly up or downregulated in both age groups (Figure 1A). By pathway enrichment analysis, approximately 1,300 gene sets were detected and about 20% of them were differentially expressed (Figure 1B). The most significantly downregulated gene sets were related to mitochondrial biogenesis, assembly of complexes, formation of super-complexes, mitochondria protein transports, expression of mtDNA-encoded genes, metabolism of fatty acids or branched-chain amino acids (BCAA), Tri-citric acid (TCA) cycle, electron transport and ATP synthesis (Figure 1C). Many upregulated gene sets were related to fibrogenesis (Figure 1C). There was high degree of consistency in the dysregulated gene sets between young and adult DCM groups (Figure 1C).

To validate the clinical simulation of this murine DCM model (Mst1-TG), we examined expression of 51 genes that were recently identified by an international panel of experts as causal genes for DCM in humans (Jordan et al., 2021). All 51 genes were identified in mouse transcriptome and approximately 60% of them were dysregulated in DCM hearts of both age groups with a higher proportion of genes upregulated (Figure 1D).

### Expression of Mitochondrial mRNAs and Transcription Promoters

**FIGURE 2:**
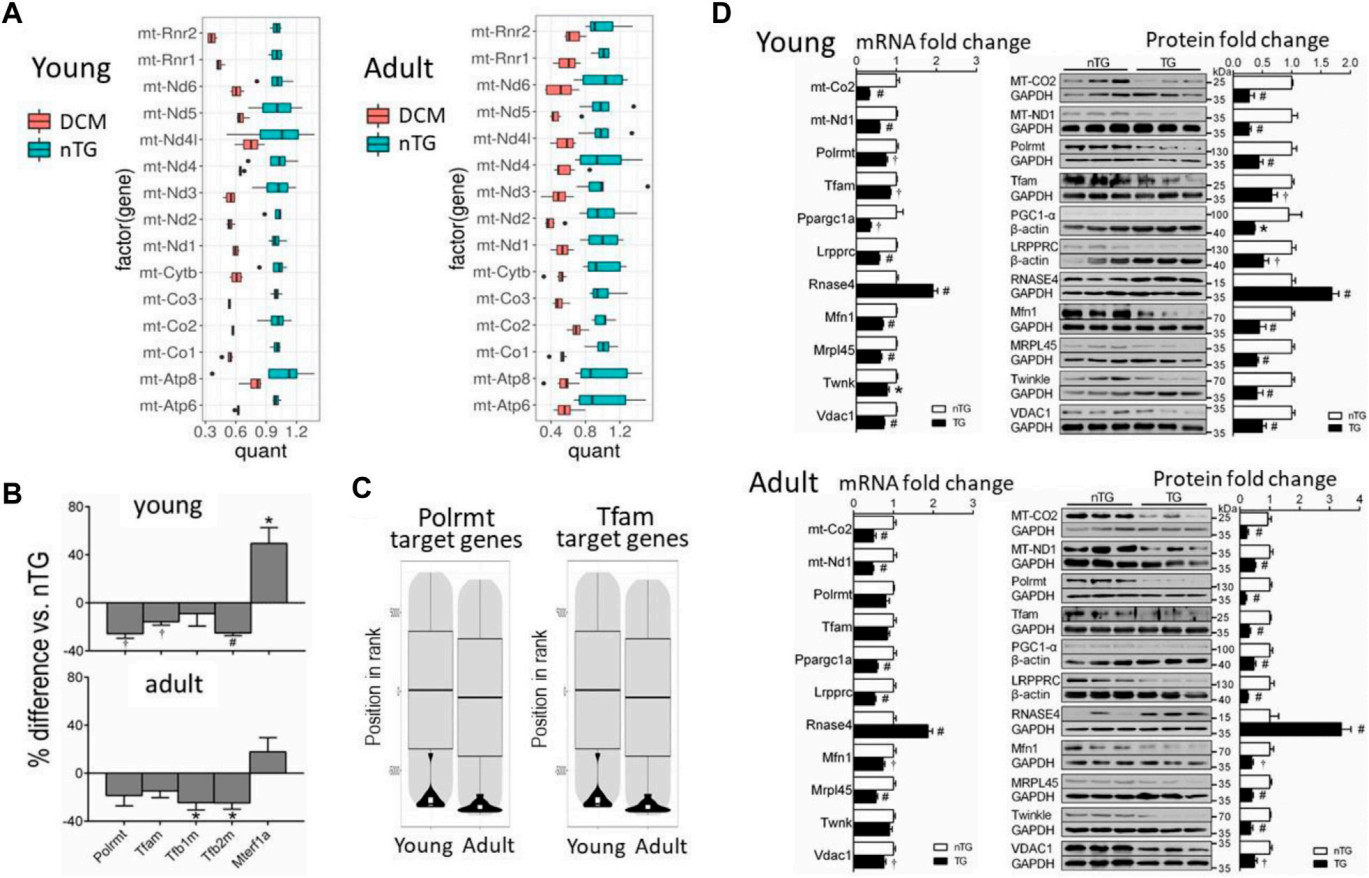
Altered expression of mitochondrial mRNAs and nDNA-encoded genes for mtDNA transcription activators or promoters in DCM hearts. **(A)** Boxplots showing downregulated expression of mitochondrial mRNAs in DCM hearts. Results were normalised to the mean of the control samples. The box height represents the interquartile range. Changes in all genes were statistically significant. Dots are outlier points defined as a data point, that is, located 1.5 times outside the interquartile range above the upper/lower quartile. **(B)** Bar graphs showing changes in gene expression of mtDNA transcription activators. Polrmt, RNA polymerase mitochondrial; Tfam, transcription factor A mitochondrial; Tfb1/2m, transcription factor B1/2 mitochondrial; Mterf1a, mitochondrial transcription termination factor 1a. **(C)** Violin plots depicting suppression of mtDNA transcription factors Polrmt target genes. **(D)** Bar graphs showing relative expression levels of selected mRNAs (RNA-sequencing) and proteins (*n* = 6 per group) for comparison. For panels B and D, *p < 0.05, ^†^ *p* < 0.01, #*p* < 0.001 vs. respective Control.

One major finding by transcriptome analysis was the profound downregulation of mtDNA-encoded 13 mRNAs in both DCM groups (Figure 2A). mtDNA transcription is regulated by mitochondrial transcription activators A or B (Tfam, Tfab1, Tfab2) that activate mitochondrial RNA polymerase (Polrmt). Expression of these genes was downregulated in DCM hearts (Figure 2B). Accordingly, Polrmt- or Tfam-target genes (i.e., mtDNA encoded genes) were markedly suppressed (Figure 2C). Expression of mitochondrial transcription termination factor 1 (Mterf1a) was higher in young DCM group. To test whether changes in mRNA expression was reflected by alterations at the protein level, we conducted immunoblotting for selected proteins, and observed reduction at protein levels of mtDNA-encoded Mt-ND1 and Mt-CO2 (cytochrome C oxidase2, by 60%–80%), and transcriptional activators Tfam and Polrmt by 35%–75% (Figure 2D). PGC-1α (peroxisome proliferator-activated receptor *γ* coactivator 1α) was implicated in promoting expression of Tfab1/2 (Gleyzer et al., 2005). In DCM hearts of both age groups, expression of PCG-1α at mRNA and protein levels was reduced by about 60% relative to nTG controls (Figure 2D).

### Enzymes for Mitochondrial RNA Processing and Mitochondrial Aminoacyl-tRNA Synthesis

**FIGURE 3:**
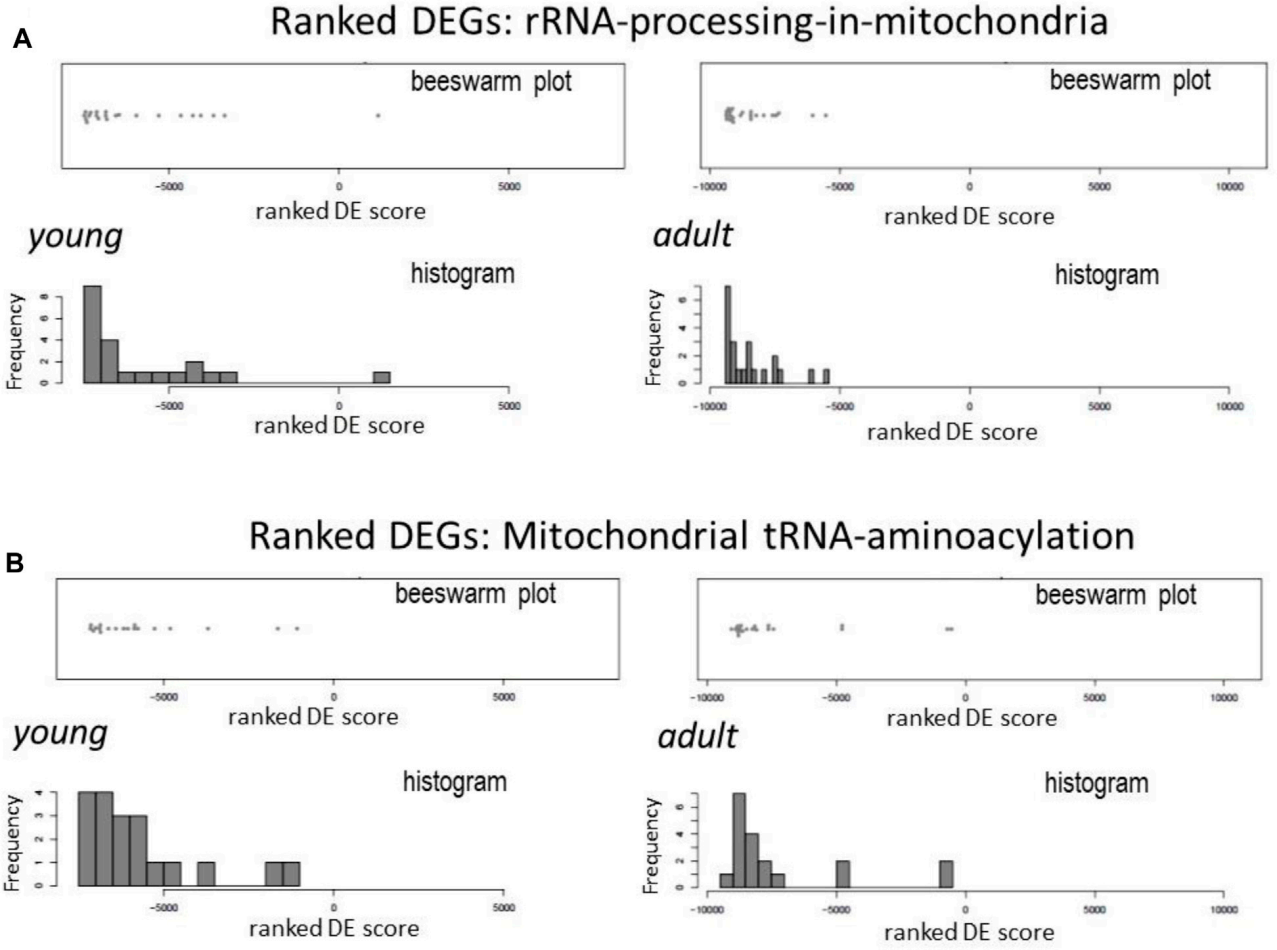
Downregulation in DCM hearts of genes coding for mt-rRNA processing and aminoacyl-tRNA synthesis enzymes. Beeswarm plots and histogram of relative changes in the expression of genes for mitochondrial RNA processing **(A)** and aminoacyl-tRNA synthases **(B)**.

mtDNA encodes two pre-rRNAs. mt-rRNAs are immediately bond by proteins to form pre-ribonucleoprotein particles to initiate the process of modifications, mostly by methylation and staged cleavage, to yield mature and functional rRNAs as the part of mitoribosome (Bogenhagen et al., 2014; Antonicka and Shoubridge, 2015). In DCM hearts, expression of genes of enzymes involved in mt-rRNA processing and mt-rRNA aminoacylation synthesis was downregulated (Figure 3A). Of these genes were mitochondrial rRNA methyltransferases (like Mrm1–3, Nsun4 and Trmt10c) and mitochondrial ribonucleases (e.g., Prorp and Elac2).

All 22 pre-tRNAs encoded in mtDNA are subjected to cleavage by ribonucleases and then modified by individual mitochondrial tRNA-synthetases to form aminoacyl-tRNAs. This process of mt-tRNA aminoacylation enables tRNA to carry amino acids to mitoribosome for protein synthesis (Sissler et al., 2017). By Reactome pathway analysis, expression of majority of genes of mitochondrial aminoacyl-tRNA-synthetases was downregulated in DCM hearts, including Aars2, Cars2, Farsb, Fars2, Hars2, Lars2, Mars2, Nars2, Ppa2, Rars2, Tars2, and Vars2 (Figure 3B).

### Expression of Mitochondrial RNA Binding Proteins

**FIGURE 4:**
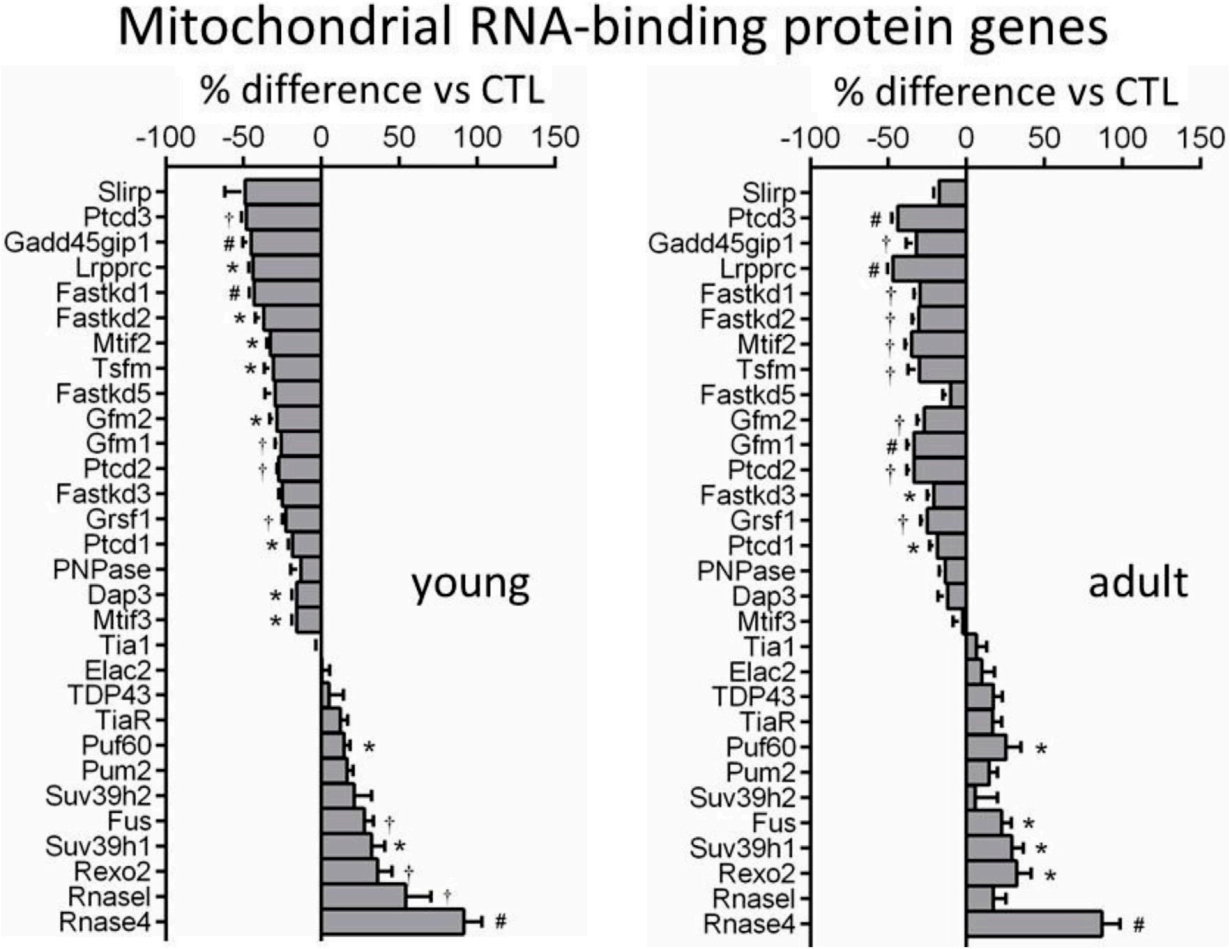
Changes in expression of genes coding mitochondrial RNA-binding proteins (MRBPs). Bar graphs showing expression levels of MRBP genes in DCM hearts of both age-groups relative to age-matched controls. *p < 0.05, †*p* < 0.01, #*p* < 0.001 vs. control.

We identified from transcriptome 30 nDNA-encoded genes of mtRBPs (Figure 4). Diverse and significant changes were observed in the expression of majority of this panel of genes in DCM relative to control hearts (Figure 4). Among downregulated genes were Slirp (SRA stem-loop interacting RNA binding protein), Gadd45GIP1 (growth arrest and DNA-damage-inducible *γ* interacting protein 1) and Dap3 (death-associated protein 3). And proteins coded by these genes are indispensable for rRNA modification and formation of mitoribosome. Downregulated also were several genes like Ptcd1-3 (mitochondrial RNase P proteins 1–3), which is implicated in mt-rRNA maturation and mitorobosome biogenesis (Perks et al., 2018), and Lrpprc (leucine-rich PPR cassette protein) known to code proteins that regulate mtRNA processing, maturation, stability and protein translation (Siira et al., 2017). Other downregulated genes were regulators of RNA transcription (Mtif, Tsfm, Gfm1). Expression was tended to be lower for polynucleotide phosphorylase (PNPase), which is an essential mitochondrial exoribonuclease involved in multiple modification processes. Another downregulated gene was GRSF1 (G-rich RNA sequence binding factor 1), known as a mtRBP required for assembly of the mitoribosome and for recruitment of mRNA and lncRNA (Antonicka, et al., 2013) (Figure 4). Another group of repressed genes belong to FAST kinase domain-containing protein family (FASTKD1-5) that are localized in mitochondria and contribute to energy balance under stressed conditions (Simarro et al., 2010). In keeping with a 50% reduction at mRNA level, protein expression of Lrpprc was significantly reduced by 40%–60% in DCM hearts of young and adult mice (Figure 2D).

Upregulated mtRBP genes included Fus (fused in sarcoma), Puf (pumillo and FBF 60 homolog), Rexo2 (RNA exonuclease 2), and RNasel and RNase4 (ribonucleases) (Figure 4). Immunoblotting revealed a 2 to 3-fold increase in protein expression of Rnase4 in DCM hearts of both age groups (Figure 2D).

### Expression of Components of Mitoribosome

**FIGURE 5:**
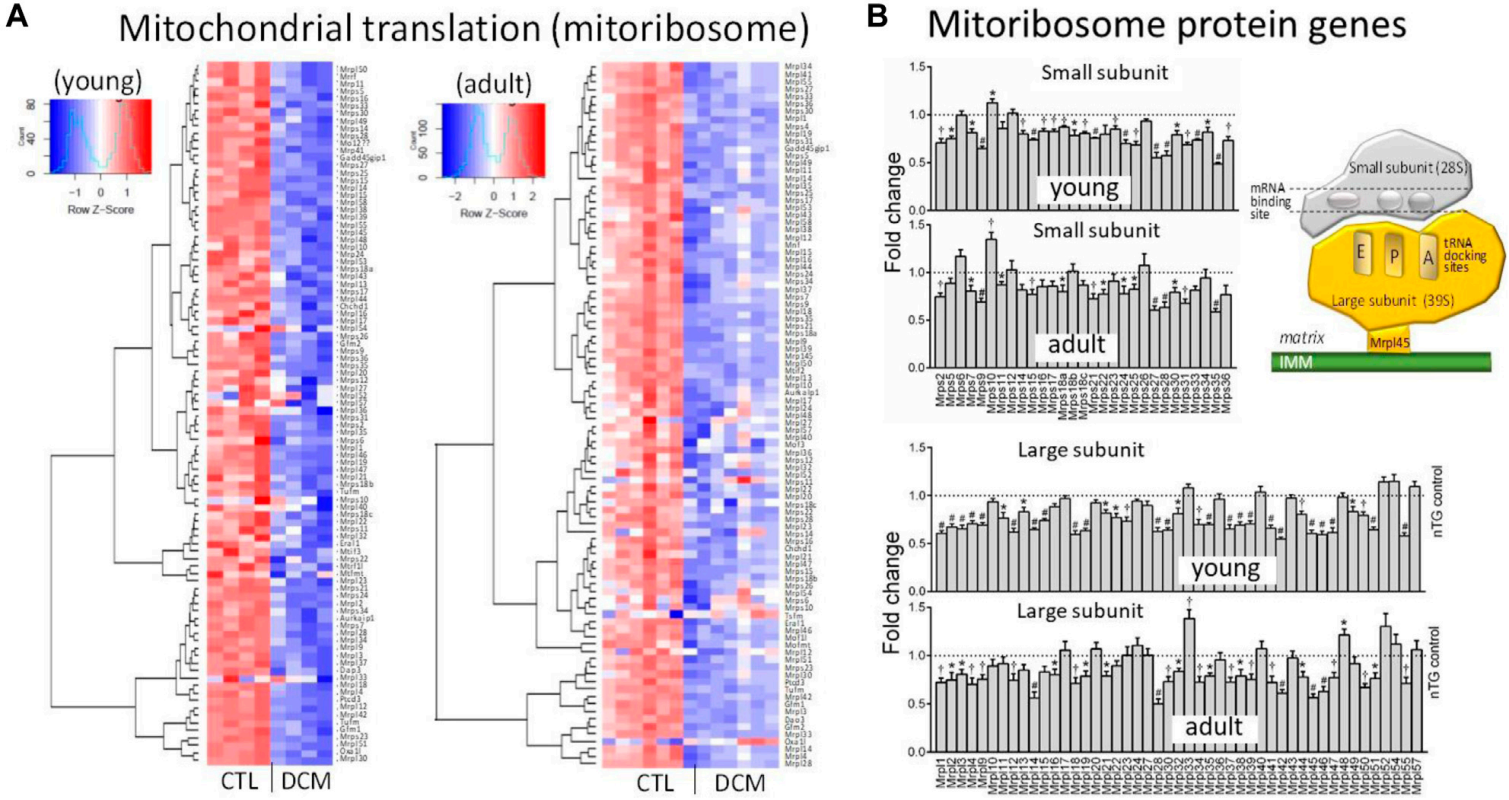
Dysregulated expression of genes for subunits of mitoribosome in DCM hearts. **(A)** Heatmap for expression of genes coding subunits of mitoribosome. **(B)** Bar graphs presenting changes in individual genes of components of small subunits (Mrps) and large subunits (Mrpl) of mitoribosome. *p < 0.05, ^†^ *p* < 0.01, #*p* < 0.001 vs. respective Control. Inserted cartoon depicting the assembly of small and large subunits of mitoribosome and the contacting site to inner mitochondrial membrane (IMM) *via* Mrpl45.

Localized within mitochondrial matrix is mitoribosome protein complex for protein translation of mtDNA-encoded 13 mRNAs (De Silva et al., 2015; Kummer and Ban, 2021). Heatmap plots showed in DCM hearts a strong trend of suppression for most genes that code small and large mitoribosome subunits (MRPS, MRPL) (Figure 5A). Further analysis of transcription level of individual genes revealed that in young DCM hearts, 75% of genes of mitoribosome subunits was significantly downregulated, and this figure was 59% in the adult DCM group (Figure 5B). In both DCM groups, several MRP genes displayed the most significant downregulation, including Mrps9/27/28/35, Mrpl14/18/42/45. Protein expression of MRPL45 was about 65% lower in DCM hearts of both age groups (Figure 2D), in consistency with a 45% reduction at mRNA level.

### Genes of Mitochondrial DNA Replicome

**FIGURE 6:**
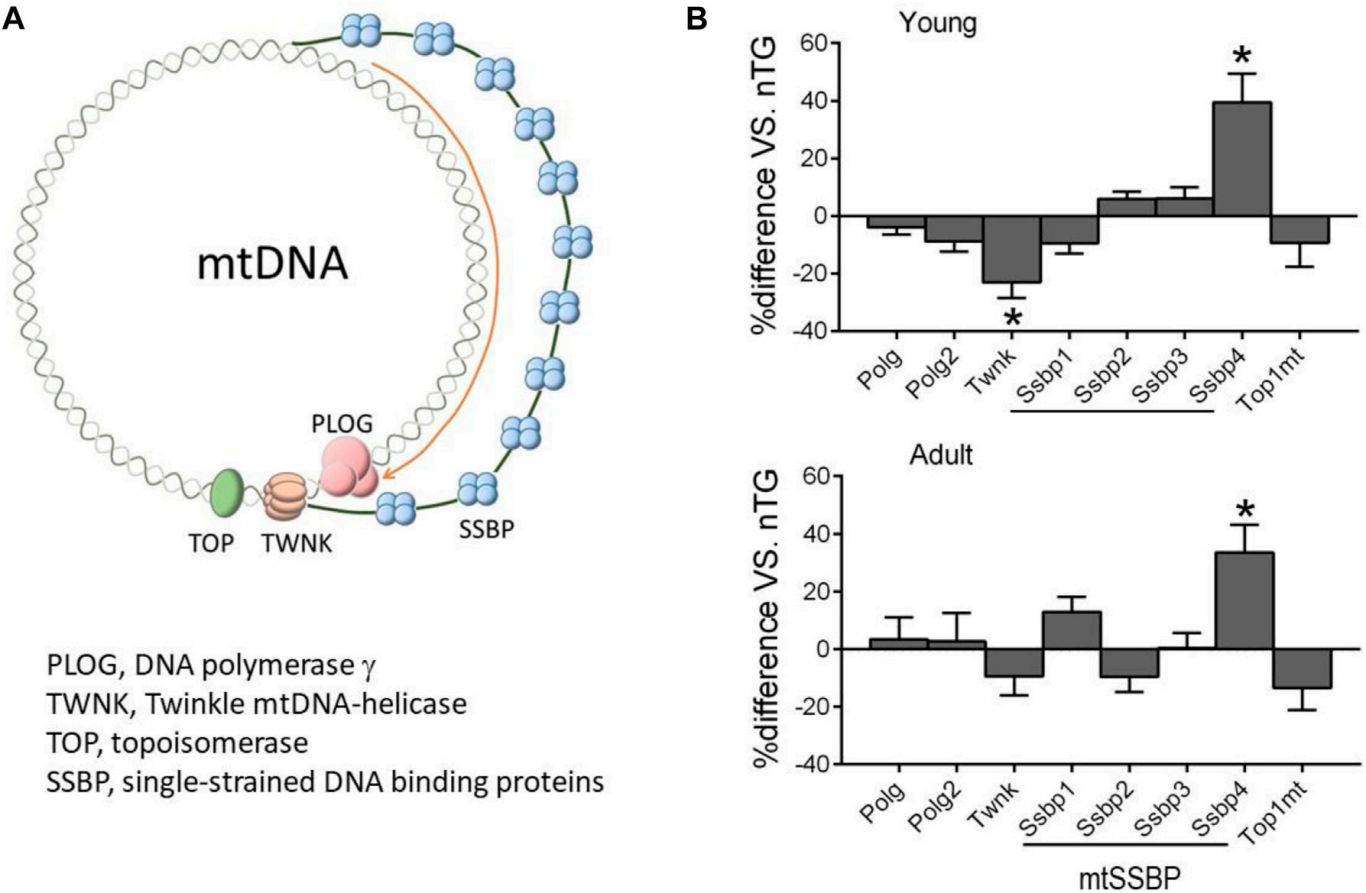
Expression of nDNA-encoded genes forming mtDNA replisome. **(A)** Diagram illustrating factors involved in mtDNA replication. **(B)** Relative expression of genes of mtDNA replisome in DCM hearts of young and adult mice. *p < 0.05 vs. respective control.

mtDNA is replicated by a nDNA-encoded protein complex forming the replisome, which consists of heterotrimer DNA polymerase *γ* complex encoded by Polg and Polg2 genes, Twinkle mtDNA helicase (Twnk), mitochondrial topoisomerase (TOPmt), and mitochondrial single-stranded DNA binding proteins (SSBP, mtSSBP) (Figure 6A) (Ikeda et al., 2015). The replication is initiated after binding by TOP1mt and Twinkle to mtDNA to unwinding the double-stranded DNA template, followed by binding of mtSSBP to single-stranded DNA for replication *via* polymerase *γ* (Figure 6B). Compared to control groups, expression of Twnk was downregulated in young but not in adult DCM hearts, while a 60% reduction in TWNK protein was observed by immunoblotting in both DCM age groups (Figure 2D). Expression of Polg, Polg2, TOP1mt, and SSBP1-3 genes was unchanged (Figure 6B) and expression of SSBP4 was significantly upregulated in both DCM groups.

### Mitochondrial Protein-Importing Machinery

**FIGURE 7:**
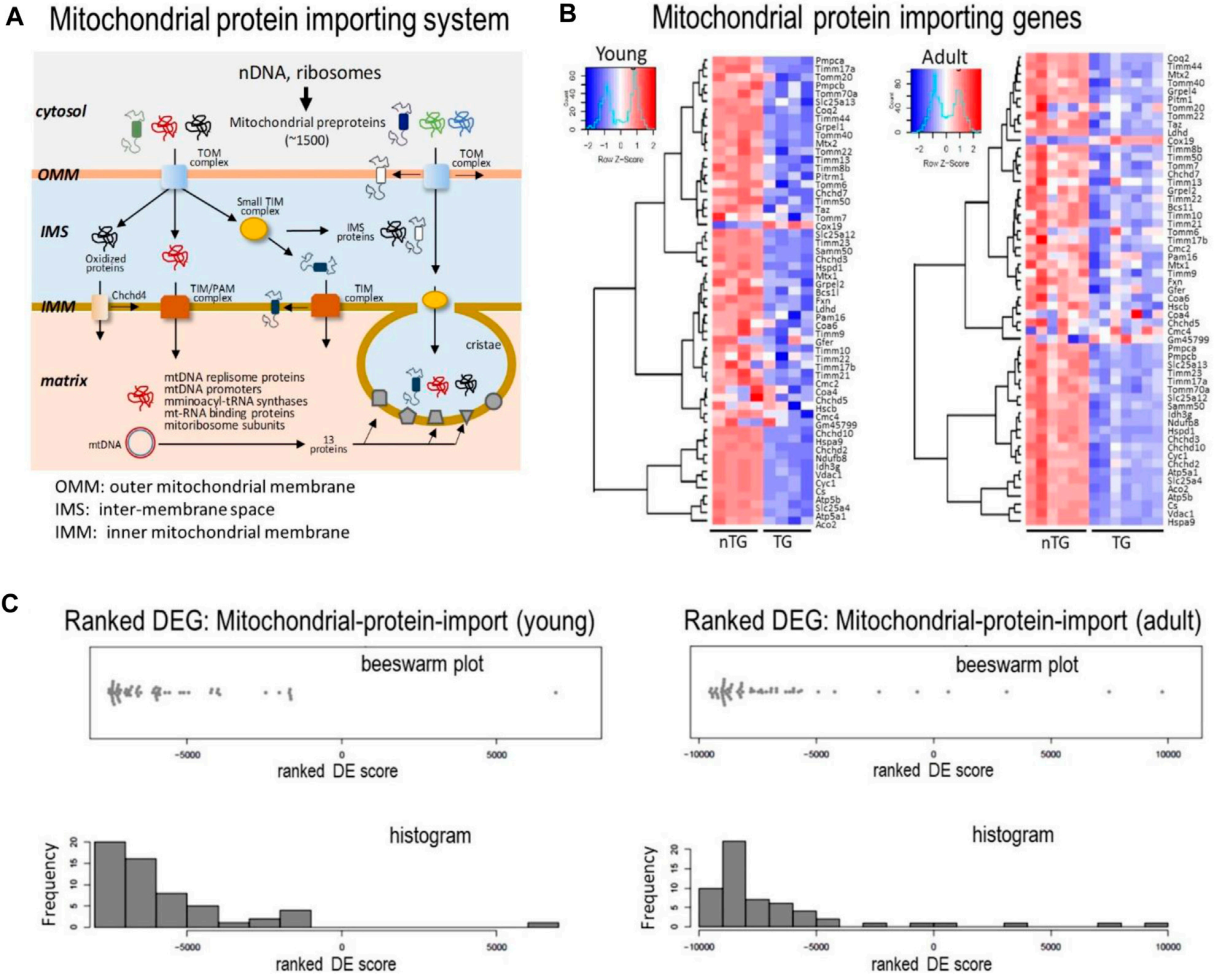
Expression levels of genes for the mitochondrial protein importing machinery. **(A)** Cartoon depicting the mitochondrial protein-importing machinery consisting of TOM and TIM complexes. Highlighted is the transportation *via* the machinery of multiple sets of proteins indispensable for mtDNA transcription and translation. Heatmaps **(B)** and Beeswarm plots and histogram **(C)** showing suppressed expression of genes coding proteins of the mitochondrial protein-importing machinery in DCM hearts from young and adult mice.

Of approximately 1,500 mitochondrial proteins, 99% are nDNA-encoded and synthesized in cytoplasm in the form of pre-proteins (Fox, 2012; Kummer and Ban, 2021). Mitochondria entry of these pre-proteins is dependent on a mitochondrial protein-import machinery (Figure 7A). Such a gateway consists of about 80 proteins forming highly specialized complexes of the translocase of outer membrane (TOM), translocase of inner membrane (TIM), sorting and assembly machinery components (SAMM) and small TIM, all participating in protein transportation, modification, folding and eventually localization (Figure 7A). In DCM hearts, there was a strong trend of downregulation for majority of genes that code components of the system (Figures 7B,C), including key subunits of TOM complex (Tomm17/20/22/40/70), TIM complex (Timm8b/10/17/21/22/23/44/50) and Samm50 (Figure 7B). Meanwhile, several genes of CHCHD family (coiled-coil-helix-coiled-coil-helix domain-containing) were downregulated in DCM hearts (Figures 7A,B). Among downregulated components of this machinery was VDAC1 gene (voltage-dependent anion channel 1, Figure 7B), which is the most abundant OMM transporting protein. In keeping with a 30% reduction in its mRNA level, VDAC1 protein was reduced by 50% in both DCM groups (Figure 2D).

## Discussion

**FIGURE 8:**
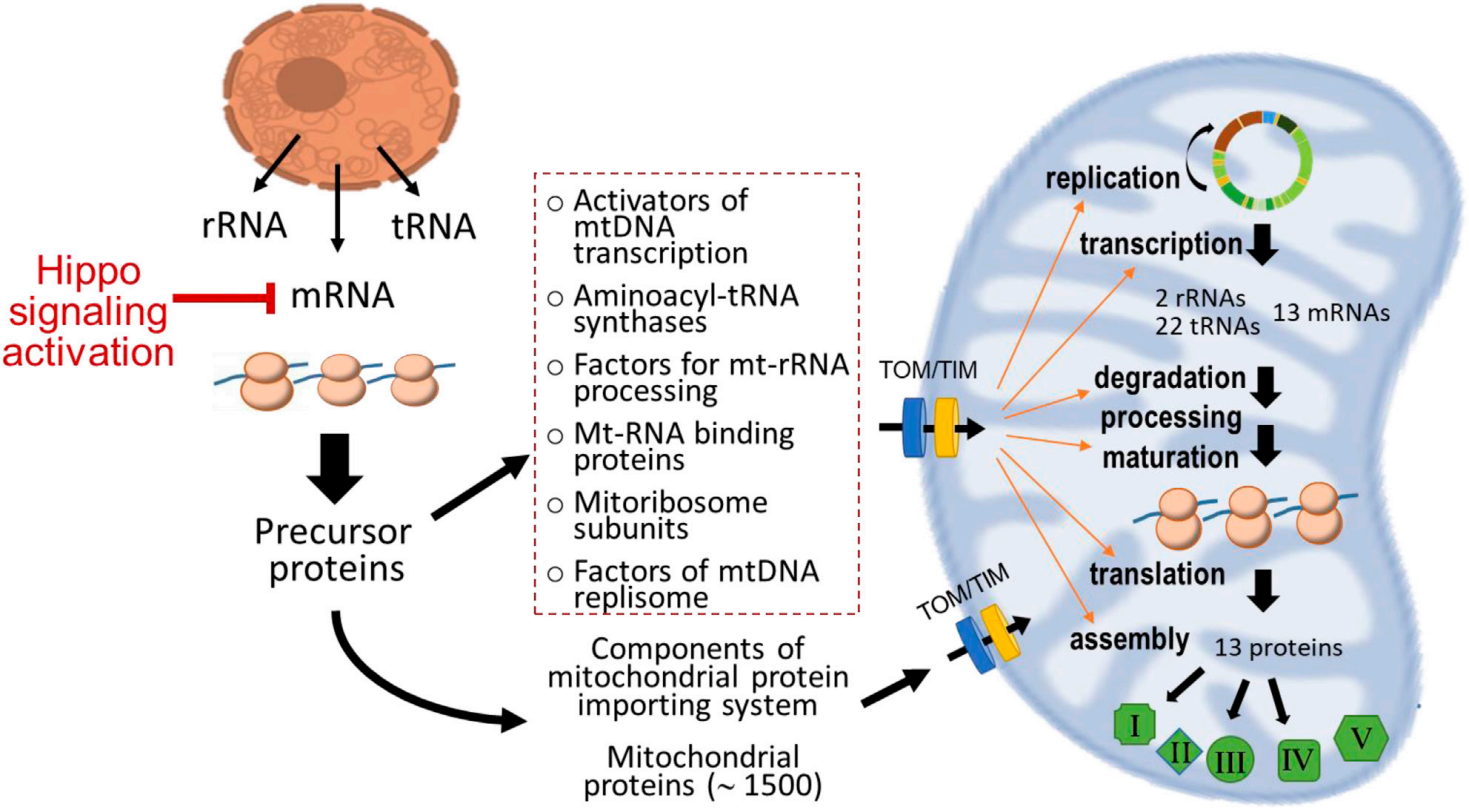
Diagram depicting classes of nDNA-encoded genes and proteins that form the nDNA-mtDNA axis. Except the 13 proteins encoded in mtDNA, all mitochondrial localized proteins are nDNA-encoded and synthesized in the cytosol. These proteins are then transported into mitochondria through the operation of specialized protein-importing machinery and ultimately localize at various sites within mitochondria. The dotted-line rectangle indicates groups of nDNA-encoded proteins forming the nDNA-mtDNA axis governing transcription and translation of mtDNA-encoded genes. In the DCM model studied, activation of Hippo signaling pathway suppressed nDNA-encoded mitochondrial genes that further interferes with this axis exacerbating mitochondrial dysfunction.

We recently demonstrated in the Mst1-TG model extensive mitochondrial genes downregulation and mitochondrial dysfunction (Wu et al., 2021). In the present study, we further illustrated in this model significant dysregulation of multiple gene sets of the nDNA-mtDNA axis. Moreover, potential dysfunction of the mitochondrial protein-importing system is implicated by downregulation of its nDNA-encoded genes, which forms another reason for protein deprivation of the nDNA-mtDNA axis, as well as numerous mitochondrial proteins in DCM hearts. These changes suggest malfunction of the nDNA-mtDNA axis contributing to mitochondrial dysfunction (Figure 8). Our transcriptome profiling highlights the interference of the nDNA-mtDNA axis by Hippo pathway signaling through transcriptional regulation of relevant genes, which becomes evident from the early phase of DCM onward.

Several features of this Mst1-TG model deserve discussion. First, there has been accumulating evidence for a causative link of enhanced Hippo signaling to DCM. Clinically, studies employing cardiobiopsy of patients with cardiomyopathy and HF revealed Hippo pathway activation and/or YAP inactivation (Chen et al., 2014; Leach et al., 2017). Experimentally, genetic models with enhanced Hippo pathway (i.e., Mst1-TG) (Yamamoto et al., 2003; Nguyen et al., 2019; Wu et al., 2021) or knockout (KO) of genes of the pathway downstream transcription factors YAP (yes-associated protein) (Xin et al., 2013) or TEAD1 (TEA-domain family member 1) (Liu et al., 2017; Liu et al., 2021), all developed similar DCM phenotypes. Here we further revealed in Mst1-TG model a number of dysregulated genes that are regarded as causal genes in human DCM (Jordan et al., 2021). All these indicate the usefulness of this mouse model in exploring mechanisms of DCM. Second, we showed downregulated mtDNA mRNAs in Mst1-TG hearts. Independent studies on Mst1-TG and TEAD1-KO mice observed extensive mitochondrial gene downregulation and metabolic dysfunction, which play a pivotal role in the onset of DCM (Liu et al., 2021; Wu et al., 2021). Human subjects carrying mutations of mtDNA-encoded genes are known to develop various types of cardiomyopathy (El-Hattab and Scaglia, 2016), which supports a mechanistic link of mitochondrial dysfunction to cardiomyopathy. Third, in Mst1-TG mice, DCM and HF phenotypes become evident as early as 3 weeks of age and progressively worsen with aging causing a shortened lifespan of 8 months (Pretorius et al., 2009; Nguyen et al., 2019; Wu et al., 2021). The similarity in dysregulated gene sets of the nDNA-mtDNA axis between young and adult DCM hearts, as shown in the current study, implies a role of abnormal nDNA-mtDNA axis in the onset and progression of DCM.

One of our key findings here was significant downregulation of all mtDNA-encoded mRNAs, the change accompanied by overt mitochondrial dysfunction in this model (Wu et al., 2021). Transcription of mtDNA-encoded genes is governed by nDNA-encoded transcriptional activators and mtRNA polymerase, Polrmt. In the DCM heart, there was a strong trend for downregulated Polrmt, finding that has not been previously reported. The activity of Polrmt is controlled by a few transcription activators (Tfam, Tfb1m, Tfb2m), and gene expression of these activators was similarly suppressed at early and severe DCM stages. This finding strongly suggests the inactivated mtDNA transcription machinery in this DCM model. Indeed, in the failing heart of humans or animals, studies have shown that expression of Tfam is progressively down (Ide et al., 2001; Watanabe et al., 2011; Kunkel et al., 2016). Earlier studies also showed that heart-restricted deletion of Tfam led to DCM and conduction blockade (Wang et al., 1999), whereas cardiac Tfam overexpression not only increased mtDNA copy number (Watanabe et al., 2011; Ikeda et al., 2015), but also was cardioprotective in the setting of chronic volume-overload (Ikeda et al., 2015).

mtDNA encodes 2 precursor rRNA and 22 pre-tRNAs. Processing of precursor rRNAs and synthesis of aminoacyl-tRNAs are essential stages for the formation of mitoribosome and synthesis of mt-mRNA encoded proteins (De Silva et al., 2015; Sissler et al., 2017). Mrm1-3, Nsun4 and Trmt10c were downregulated in our DCM hearts, and these genes code mitochondrial rRNA methyltransferases, and downregulation of these genes would be expected to compromise the processing and modification of mt-rRNAs. Our Reactome pathway analysis showed that expression of almost all aminoacyl-tRNA-synthetase genes were markedly downregulated in young and adult DCM heart. After transcription, pre-tRNAs are cleaved by ribonucleases and then catalysed by individual mitochondrial aminoacyl-tRNA-synthetases, conjugation with individual amino acids to form respective cognate tRNA molecule. Such a process of mt-tRNA aminoacylation enables tRNA to carry amino acids to mitoribosome for protein synthesis. Thus, downregulated aminoacyl-tRNA synthetases likely interfere with protein synthesis in mitochondria. There have been clinical reports of mitochondrial hypertrophy or DCM associated with homozygous mutation in AARS2 gene (Gotz et al., 2011; Fine et al., 2019; Nielsen et al., 2020) in addition to mitochondrial disorders involving the central nervous system (Fine et al., 2019).

About 30 genes of mitochondrial RNA binding proteins (MRBPs) were identified and diverse changes in their expression were observed from downregulation by 50% (like Ptcd, Gadd54GIP1, Prpprc, Fastk, Grsf1), unchanged, to significant upregulated (e.g., Fus, Rexo2, Rnase4). Mt-mRNAs are known to bind with multiple MRBPs (Bogenhagen et al., 2014; Barchiesi and Vascotto, 2019). MRBPs are at the central of post-transcriptional gene regulation, coordinating processing, storage, stability and degradation of mt-rRNA. Of downregulated genes, Lrpprc and Slirp code for proteins that interact and bind to mt-mRNA to protect mt-mRNAs against degradation and ensure stability of mitochondrial transcriptome (Lagouge et al., 2015; Siira et al., 2017). *In vitro* studies have shown that Lrpprc knockdown is associated with lower level of mt-mRNA like mt-CO1/3, and suppressed mitochondrial translation (Sasarman et al., 2010). Moreover, mutation of Lrpprc is linked to the Leigh Syndrome, a neurological disorder due, in high percentage of patients, to mutations of mitochondrial DNA (Cruz et al., 2019). In DCM hearts, expression of all 5 variants of Fastkd family was consistently suppressed. Fastkd members are known to present in mitochondria and are dispensable for processing and maturation of certain mt-mRNAs and cellular respiration, and that Fastkd2 gene mutation is linked to mitochondrial encephalomyopathy (Simarro et al., 2010; Antonicka and Shoubridge, 2015). MRBP consists a panel of ribonucleases and changes in the expression of these ribonucleases in heart disease is unknown. We identified upregulation of Pum2, Puf60, and Fus (fused in sarcoma) known as translation repressors (Ravanidis and Doxakis, 2020). Mitochondria-localized Fus has been shown to induce mitochondrial damage (Deng et al., 2015; Tsai et al., 2020). Other upregulated genes like PNPase (polynucleotide phosphorylase), Rexo2 (RNA exonuclease 2), RNase L/4 (ribonuclease, latent/4) and RNase P (Ribonuclease P) code enzymes that cleavage nucleotides and mRNAs (Ravanidis and Doxakis, 2020). In the DCM heart, RNase4 protein level increased by 2–3 fold and further study is warranted to explore its significance in DCM. PNPase is an essential mitochondrial exoribonuclease with multiple function, and PNPase deletion results in loss of mtDNA (Schmidt S. P. et al., 2010; Englmeier et al., 2017). Upregulated Suv39h1 gene belongs to DNA-methyltransferases and the role of this change remains unclear although certain DNA-methyltransferases have been found in mitochondria (Sharma et al., 2019).

Throughout evolution, mitochondria keep their own genome for *in situ* transcription and ultimately synthesis of 13 polypeptides to be integrated into OXPHOS complexes localized at IMM. Mitoribosomes in the matrix are assembled by approximately 80 nDNA-encoded subunits and 2 rRNA molecules transcribed from mtDNA forming macromolecular machinery of small subunit (SSU) and large subunit (LSU) (Schmidt O. et al., 2010; Englmeier et al., 2017). We observed in the DCM heart diverse changes in gene expression of mitoribosomal subunits with a high proportion of genes downregulated. Mrpl45 forms the single major contact site anchoring mitoribosome to IMM and such a structural link is critical for translocating newly synthesized proteins to respective OXPHOS complexes (Englmeier et al., 2017). Importantly, we found that expression of Mrpl45 at mRNA and protein levels was markedly suppressed. It is likely that suppressed expression of this class of genes would compromise assembly of functional mitoribosome, ultimately affecting protein expression of mt-mRNAs in failing heart, as shown in the current and previous studies (Kummer and Ban, 2021).

There has been few report on alteration in diseased heart of either expression of genes responsible for mtDNA replication, or mtDNA copy number. Here we observed in DCM hearts of young and adult mice, suppression of mitochondrial specific DNA helicase Twinkle at mRNA and/or protein levels. Ide et al. (2001) reported in mice that cardiomyocyte-restricted overexpression of Twinkle led to a 2-fold increase in mtDNA copy number, and that when subjected to either chronic pressure- or volume-overload, Twintle-overexpressed mice were protected against ventricular remodelling, dysfunction and fibrosis (Tanaka et al., 2013; Ikeda et al., 2015). In both DCM groups, expression of single-stranded DNA-binding protein (mtSSB4) was upregulated, which might represent a compensatory response in the setting of DCM and might explain in part our previous finding in DCM hearts a 20% increase in mitochondria density (Wu et al., 2021).

Nearly all mitochondrial precursor proteins are synthesized in the cytosol and then transported into mitochondria *via* the highly specialized protein-importing system (Fox, 2012). Consisting of approximately 7% of total mitochondrial proteins (Schmidt O. et al., 2010), this system operates through sophisticated supercomplexes of translocases (TOMs, TIMs) with functionality from preprotein recognition by receptors at OMM, specific TOM-preprotein binding, translocation, processing and maturation, to assembly into functional complexes (Figure 8) (Schmidt O. et al., 2010; Englmeier et al., 2017; Kummer and Ban, 2021). TOM complex transports proteins into OMM or IMS, and coordination is required for TIM complex and soluble small TIMs for further transportation from IMS to IMM or the matrix (Schmidt O. et al., 2010). In DCM hearts of both ages, downregulation is evident for multiple gene families of the importing system, including Tomm, Timm, Samm, and Chchd. As we are aware, this is the first study to reveal such extensive downregulation of genes of the protein-importing machinery in DCM hearts. We noticed downregulated genes forming TOM core (Tom40/22/5/6/7) or TOM receptors (Tom20/22/70) (Wiedemann and Pfanner, 2017). We also observed downregulation of multiple Timm genes coding proteins that form TIM integral (Tim23/17/55) or TIM peripheral components (Tim50/21) (Endo et al., 2003). Expression of Samm50 was also suppressed. Meanwhile, downregulated are several members of CHCHD family Chchd2/3/10/13. In addition to formation and stabilization of cristae, CHCHD family members, localized in IMS or IMM, are critical for importation and/or the post-import folding of substrates by catalysing formation of disulfide bonds (Modjtahedi et al., 2016). Proteins involving in mtDNA transcription and translation all need to be transported through OMM, IMS and IMM to reach the matrix, where mtDNA and mitoribosome are localized (Figures 7A, 8). Thus, it is postulated that transportation and precise localization of these proteins are more likely to be compromised if the importing system is dysfunctional. Indeed, deletion of individual components of this importing system has been shown to influence not only assembly of respiratory complexes, but also synthesis of mtDNA-encoded proteins as well as mtDNA homeostasis (Ott et al., 2012; Wiedemann and Pfanner, 2017).

Study limitations deserve some comments. We performed protein validation of a selected panel of genes with results showing good consistency. However, we did not perform further experiments along the directions as indicated by transcriptome for causative evidence. This was in part due to the fact that our transcriptome profiling revealed an excessive number of dysregulated genes that deserve further investigation by techniques including genetic interventions. However, these mitochondrial proteins of interest usually form functional complexes, which makes selection of target(s) for genetic interventions a challenge. Future study is also warranted to further elaborate these changes by employing combined RNA-sequencing and mitochondrial genome transcriptome, together with specific mitochondrial functional assays. The role of non-coding RNA in the dysregulated transcriptome also deserves further research. Nevertheless, our findings provide useful reservoir of information for future investigation on the nDNA-mtDNA axis in the setting of heart disease.

In conclusion, transcriptome analysis revealed in the DCM mouse model significant dysregulation, mainly downregulation, of nDNA-encoded genes for proteins involved in mtDNA transcription and translation as well as mitochondrial protein-importing. Our results from the transcription level highlight the complexity of the operation of nDNA-mtDNA axis and implicate a role of the Hippo signaling pathway in mediating malfunction of the nDNA-mtDNA axis contributing to the onset of mitochondrial dysfunction and DCM.

## Data Availability

The datasets presented in this study can be found in online repositories. The RNA sequencing data were uploaded to the Gene Expression Omnibus (GSE106201).

## Glossary

Chchd: coiled-coil-helix-coiled-coil-helix domain containing
Dap3: 28S ribosomal protein S29, mitochondrial
DEG: differentially expressed gene
Fastkd: FAST kinase domain-containing protein family
Fus: fused in sarcoma
Gadd45GIP: growth arrest and DNA-damage-inducible protein GADD45 alpha
Gfm1/2: elongation factor G 1, mitochondrial
Grsf1: G-rich RNA sequence binding factor 1
Lrpprc: leucin-rich pentatricopeptide repeat (PPR)-containing
Mgmt: O-6-methylguanine-DNA methyltransferase
Mrpl, Mrps: mitochondrial ribosome protein large or small subunit
Mrm1-3: mitochondrial rRNA methyltransferases1-3
mtERF: mitochondrial transcription termination factor
Mtif1-3: metal regulatory transcription factor 1
mtPAP: mitochondrial poly-A polymerase
MRBP: mitochondrial RNA binding proteins
Nlr: nucleotide-binding Leucine-rich repeat family receptor
OXPHOS: oxidative phosphorylation
Pgc-1α: peroxisome proliferator-activated receptor gamma coactivator 1α
Pnpase,: polynucleotide phosphorylase
Polg: DNA polymerase gamma, mitochondrial
Polrmt: RNA polymerase; mitochondrial
Ptcd: pentatricopeptide repeat domain, mitochondrial
Pup: pumillo and FBF homolog family
Pum2: pumilio homolog 2
Rexo2: RNA exonuclease 2
Rnase l/4: ribonuclease Latent/4
Slirp: SRA stem-loop interacting RNA binding protein
SSBP: single-stranded DNA-binding protein, mitochondrial
TEAD: TEA-domain family member
Tefm: mitochondrial transcription elongator factor
Tfabm: transcription factor B, mitochondrial
Tfam: transcription factor A, mitochondrial
TIM: translocase of inner mitochondrial membrane
TOM: translocase of outer mitochondrial membrane
Top1: topoisomerase 1
Tsfm: Ts translation elongation factor, mitochondrial
Twnk: twinkle mitochondrial DNA helicase
YAP: yes-associated protein
